# Supplementary material for: RAFFT: Efficient prediction of RNA folding pathways using the fast Fourier transform
Source: PLoS Comput Biol. 2022 Aug 26;18(8):e1010448. doi: 10.1371/journal.pcbi.1010448 (PMC9455880; doi:10.1371/journal.pcbi.1010448)
Supplement: S1 Appendix — Additional numeric experiments for comparing other folding tools and execution times. (PDF) [file pcbi.1010448.s001.pdf]

Supplementary material: RNA fast-folding paths for the prediction of secondary structures and folding dynamics

Vaitea Opuu, Nono S. C. Merleau, Vincent Messow, and Matteo Smerlak

Max Planck Institute for Mathematics in the Sciences, Leipzig, Germany

August 2, 2022

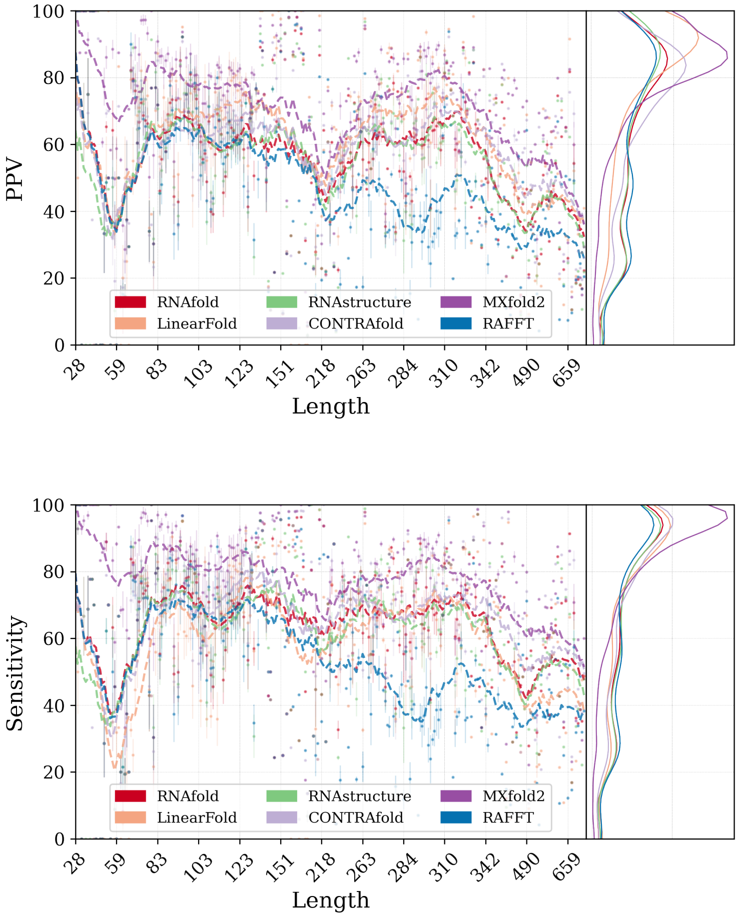

Fig A: **Performance by means of PPV and sensitivity of all tested methods.** The methods displayed are as shown in Figure 1 of the main text. `MXfold2` (ML) is herein included. In the main text, the selection of tools was reduced to `RNAfold` as a representative for thermondynamics based methods and `MXfold2` for machine-learning based methods respectively.

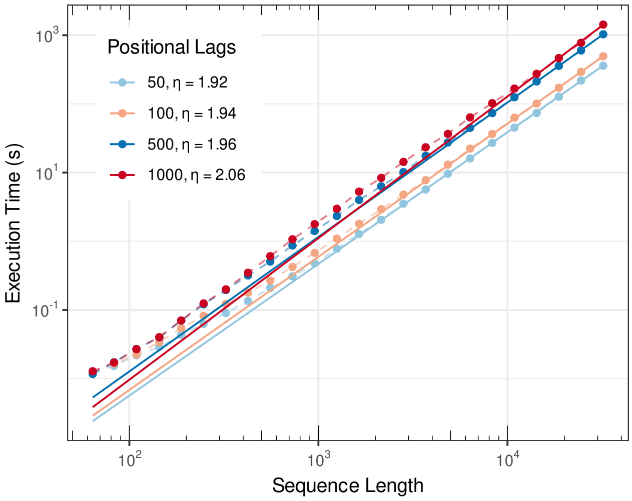

Fig B: **Impact of the number of positional lags n on runtime complexity.** For a corresponding length, we generated 30 random sequences, and averaged their execution times. Solid lines indicate the estimated time complexity $O(L^{\eta})$ where η is obtained with a non-linear regression on these average execution times for different numbers of positional lags n.

Table A: **Average performance displayed in terms of PPV and sensitivity.** The metrics were first averaged at fixed sequence length, limiting the over-representation of shorter sequences. The first two rows show the average performance for all the sequences for each method. The bottom two rows correspond to the performances for the sequences of length $\leq$ 200 nucleotides.

	`RNAfold`	`LinearFold`	`RNAstructure`	`CONTRAfold`	`MXfold2`	`RAFFT`
			All sequences			
PPV	55.9	60.6	54.7	58.4	70.4	47.7
Sensitivity	63.3	58.9	61.5	65.2	77.1	52.8
			Sequences with lengths $\leq$ 200			
PPV	59.5	63.2	58.2	60.5	76.7	57.9
Sensitivity	65.5	59.4	63.8	65.9	82.9	63.2

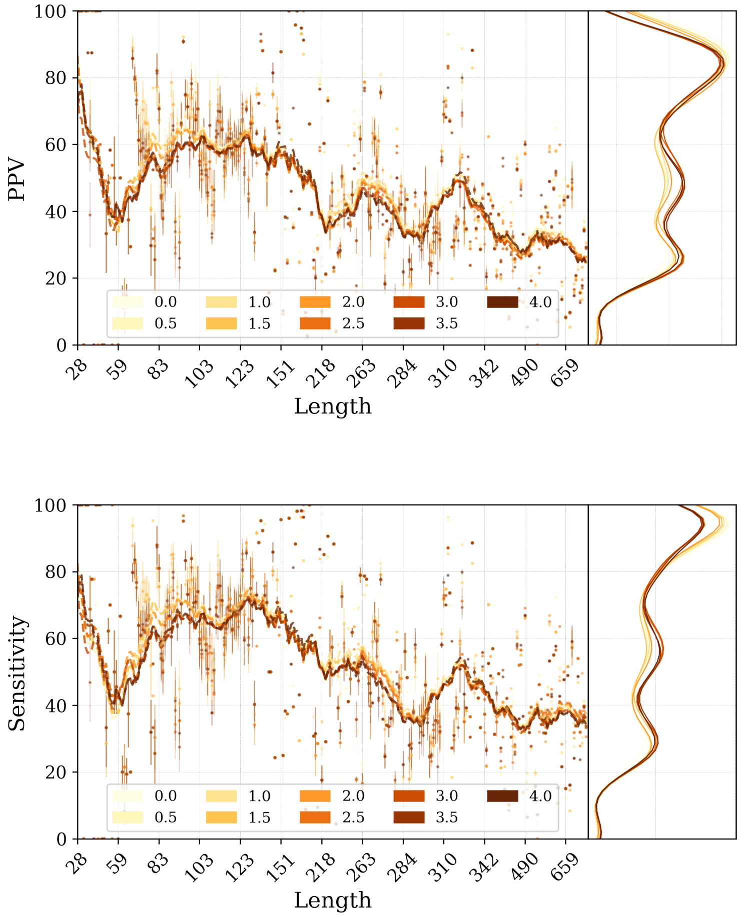

Fig C: **Predictive performance of RAFFT with various values of minimum energy contribution required for loop formation.** Positive values for this parameter causes RAFFT to accept destabilizing loops, therefore being less greedy than per default. The performance of RAFFT was not observed to be positively affected by allowing sub-optimal loop formation.

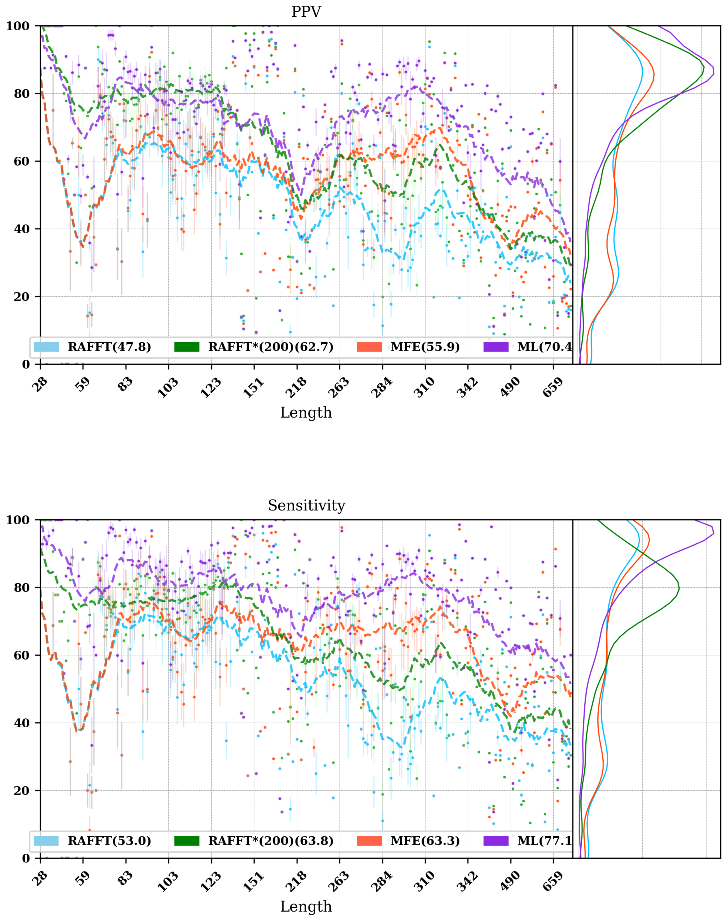

Fig D: **Positive predictive values and sensitivity results.** RAFFT (blue) displayed the best energy found. RAFFT*(200) shows the best score found among 200 saved structures. Left pans show the density (sequence-wise) of the accuracy measures.

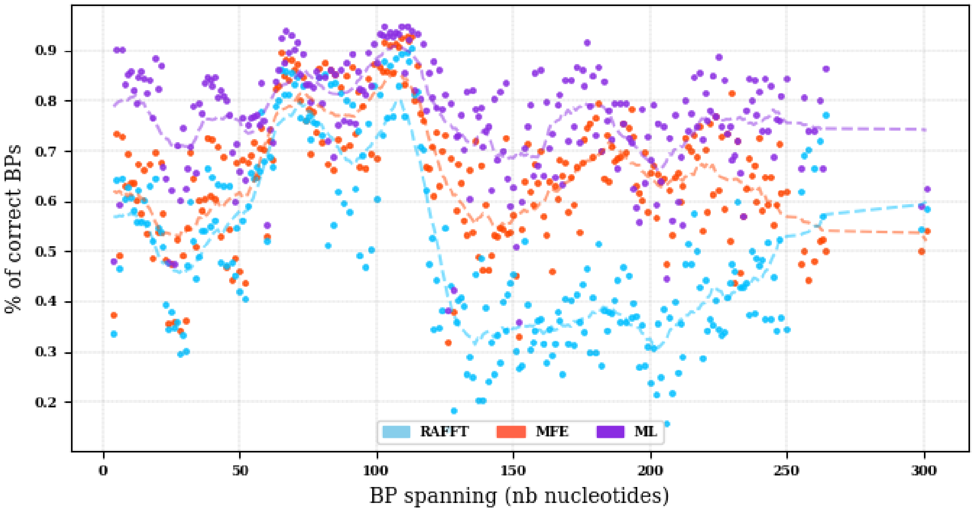

Fig E: **Base pair spanning: It shows the percent of base pairs predicted found in the known structures per number of nucleotides between them.**

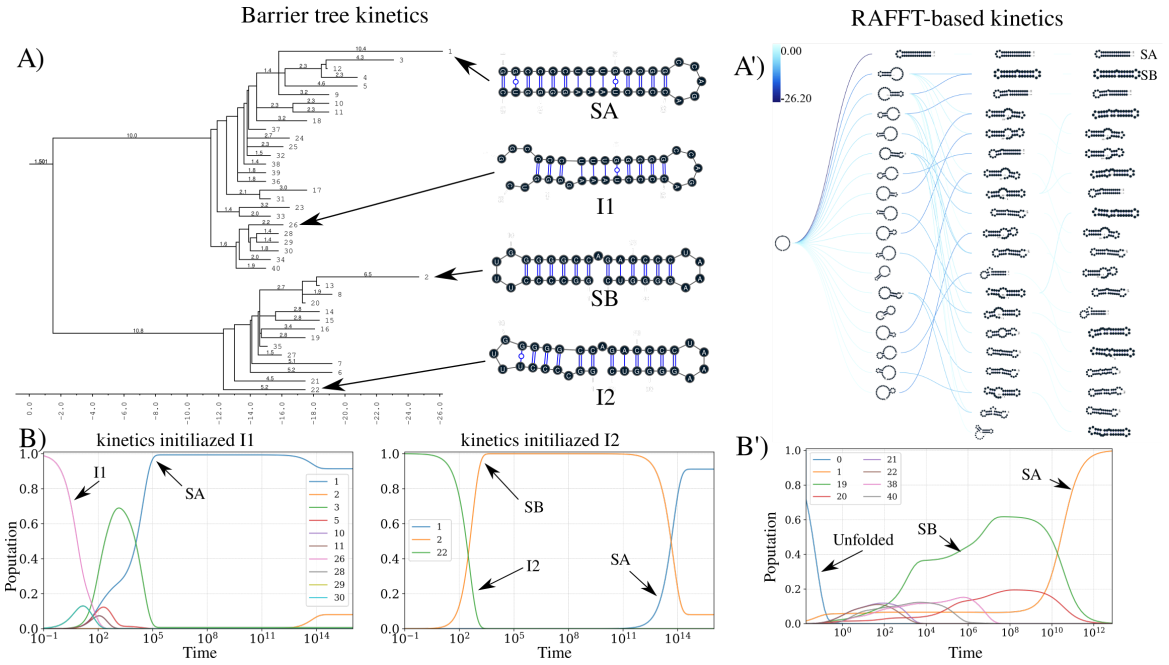

Fig F: `RAFFT` ***vs*** `Treekin`**: folding kinetics of a bi-stable RNA sequence.** (A) Barrier tree for the bi-stable example sequence. The local minima and the corresponding barriers are computed from the complete enumeration of the structure space. The bi-stability is visible on the barrier tree through the two branches separated by a high barrier. (B) Folding kinetics trajectories. The left plot shows the folding dynamics starting from a population with I_1, and the right size is the kinetics when the population is initialized in structure I_2. When starting from I_1, S_A is quickly populated; starting from I_2, the bi-stability is more apparent. (A') Fast-folding graph using `RAFFT`. A maximum of $N = 20$ structures are stored in a stack at each step and overall 46 distinct structures are visited. (B') Folding kinetics trajectory obtained from the fast-folding graph (indices are different from the barrier tree indices). The dynamics starts with a population with only unfolded structure, and slowly, S_B is populated and gets trapped for a long time before the MFE structure S_A becomes populated.

As a second illustrative example, we applied both kinetic models to the classic bi-stable sequence `GGCCCCUUUGGGGGCCAGACCCCUAAAGGGGUC`. For `Treekin`, we first sampled the whole space of 20×10^3 sub-optimal structures from the unfolded state to the MFE structure, and from that set, 40 basins were also computed using `barriers`. The barrier tree in Figure F shows the bi-stable landscape, where the two deepest minima are denoted S_A and S_B. As in the first application, we also chose two initializations with the structures denoted I_1 and I_2 in Figure FA and FB. Secondly, we simulate the kinetics starting from the two initial conditions (See Figure FB). When starting from I_2, the slow-folding dynamics is visible: S_B first gets kinetically trapped, and the MFE structure (S_A) only takes over later on. For our kinetic ansatz, we started by constructing the fast-folding graph using `RAFFT`, consisting of only 46 distinct structures. The resulting kinetics, shown in Figure FB' was found qualitatively close to the barrier kinetics initialized with structure I_2. Once again, with few as 48 structures, our proposed kinetic ansatz can produce complete folding dynamics starting from a population of unfolded structure.

Kinetic comparison

According to the RNA structure thermodynamics, one RNA molecule can adopt a structure s with probability $p(s) \propto \exp(-\beta\Delta G(s))$, where β is the inverse thermal energy (mol/kcal). To measure the quality of the ensemble of structures proposed by our method, we measured: (1) the average probability of each structures in the ensemble, then (2) the diversity of these structures.

The probability coverage PC given by $PC(s) = \frac{1}{|\Omega|}\sum_{s\in\Sigma} p(s)$. Ω is the ensemble of structures sampled by a given method. We compared, for various random sequences, the probability coverage to methods based on Boltzmann sampling [**?**, **?**]. We generated ensembles of $10^2, 10^3$, and 10^4 structures per sequence denoted respectively SB100, SB1K, and SB10K. In addition, we also compared to `RNAxplorer`, a tool also based on a biased Boltzmann sampling.

All structures are represented in the dot-bracket notation. In the dot-bracket notation, one structure has $ss = \{(, .,)\}$ symbols at each position. Given these three symbols, we propose the following positional entropy measure $S = \frac{1}{L}\sum f_i(ss) \times \log(f_i(ss))$, where $f_i(ss)$ is the frequency of a symbol ss at position i in the ensemble of structure proposed.

Figure H shows the probability coverage and the positional entropy measure per method. It shows comparable sampling performances for fairly size sequences ($\approx 10^2$ nucleotides); and a comparable diversity.

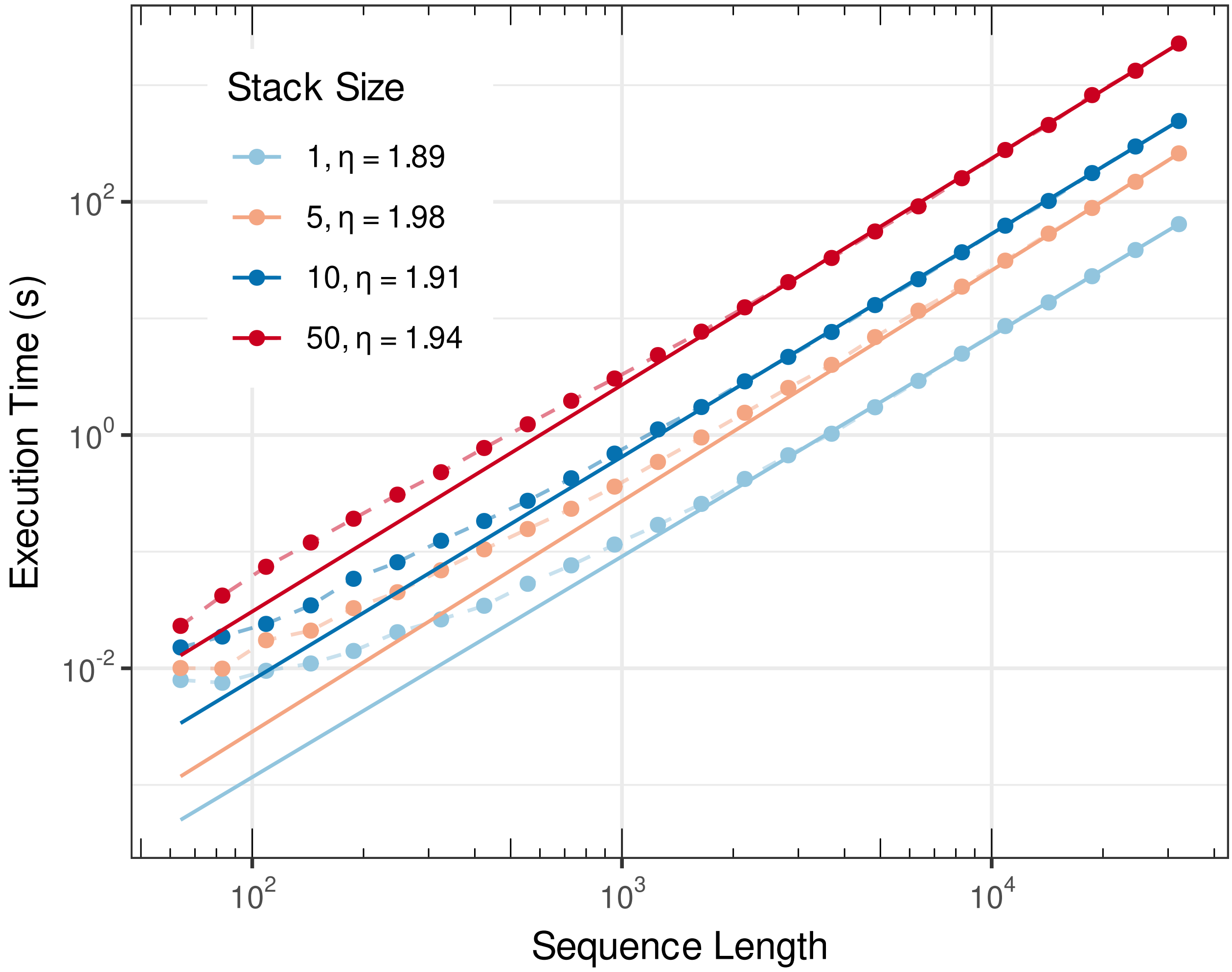

Fig G: **Impact of the number of stack size N on runtime complexity.** For a corresponding length, we generated 30 random sequences, and averaged their execution times. Solid lines indicate the estimated time complexity $O(L^{\eta})$ where η is obtained with a non-linear regression on these average execution times for different stack sizes N.

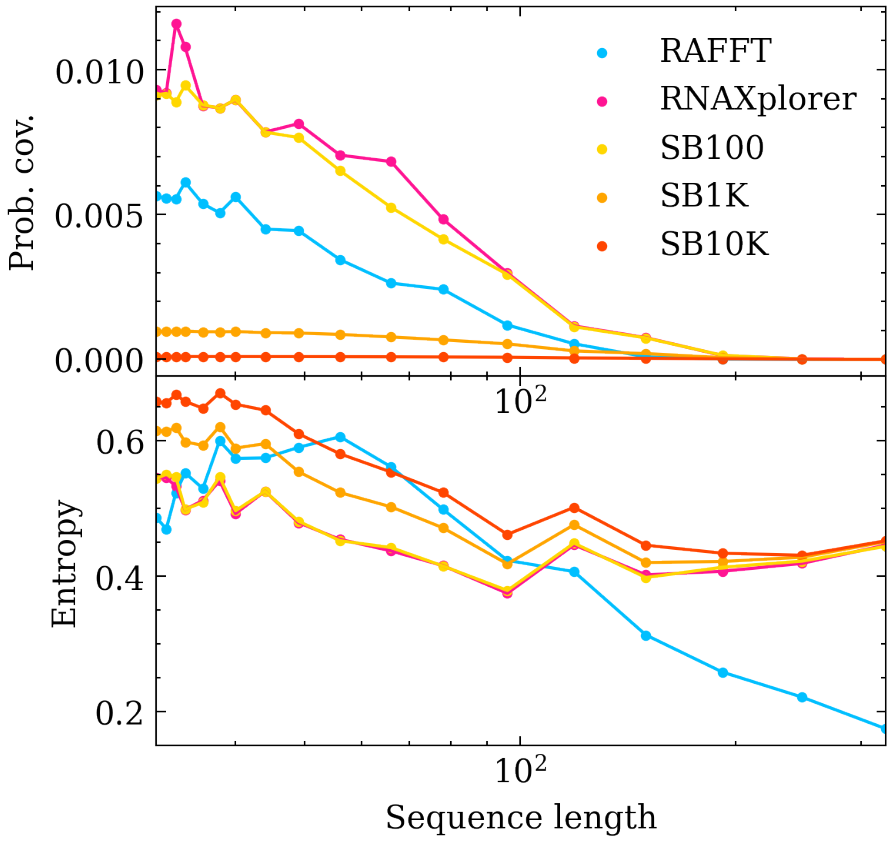

Fig H: **Structure ensemble characterization.** The upper part shows the average probability summed over the ensembles of structures predicted per sequence with different methods. The bottom part shows the average positional entropy of structures using the dot-bracket notation.
