# Supplementary material for: RAFFT: Efficient prediction of RNA folding pathways using the fast Fourier transform
Source: PLoS Comput Biol. 2022 Aug 26;18(8):e1010448. doi: 10.1371/journal.pcbi.1010448 (PMC9455880; doi:10.1371/journal.pcbi.1010448)
Supplement: Attachment — Submitted filename: review.pdf [file pcbi.1010448.s002.pdf]

Replies — RAFFT: Efficient prediction of RNA folding pathways using the fast Fourier transform

July 20, 2022

We thank both reviewers for their constructive comments that helped improve our manuscript. The modifications proposed are given below in red whereas our direct reply and justifications to the comments are given in blue.

Reviewer 1

Reviewer 1's Description

Reviewer #1: In this article, the authors describe a new approach to prediction of RNA secondary structure. Authors have performed a nice literature survey of the previously developed methods and tools before proposing their new approach. The folding algorithm and kinetic ansatz has been developed and described very nicely by the authors. Although ML-based approaches have shown performances much better than RAFFT, the authors have acknowledged the advantages and disadvantages of the method in details. Overall the paper appears to be very well-written and holds the standards of the scientific presentation highly. The paper is very well suited for the journal and will be interesting to the readers. The reviewer only has minor comments.

Comment 1 — Page 1, "... structures; evidence suggests..." Please add the citation.
Reply — R1 recommended giving a reference of evidence for the hierarchical behavior of the RNA folding process where it starts with the secondary structure to the tertiary structure. As an answer, we added the reference to one work from Liphardt *et al*, where they showed by applying unfolding mechanical forces to one RNA molecule that there is a gap in energy contribution between secondary and tertiary structures, supporting the crucial importance of secondary structure over the tertiary.
Modification — This separation of time scales justifies focusing on the prediction of secondary structures; evidence from molecular mechanical stretching experiments [1]

Comment 2 — Figure 1 - the caption could use more details. Although it is introductory figure, it could also be used to summerize the folding algorithm and kinetic ansatz that will keep readers interested.
Reply — R1 asked for a more detailed description of the framework in the caption. As an answer, we added in the caption of Figure 1 details of the algorithm: the numerical encoding; the step in which the FFT is used.
Modification — (1) The sequence of nucleotides of the RNA molecule is first encoded numerically to facilitate its treatment. (2) The numerically encoded sequence is analyzed using the FFT to detect stems quickly, such that stems are added iteratively to form parallel folding paths. (3) The folding pathways form a graph that connects potential intermediate secondary structures. Starting with the unfolded structure, our kinetic ansatz predicts a complete folding trajectory (*i.e.* when the RNA molecule adopts a specific structure.)

Comment 3 — RAFFT - It would be nice if the authors can actually provide a term and abbreviation connection somewhere they first use it.
Modification — RAFFT (RNA folding Algorithm with Fast Fourier Transform)

Comment 4 — Page 3, please merge the sentences "we compute free energy change ..." and "Here, free energies were computed"
Modification — we compute the free energy, using the ViennaRNA package API [2], change associated with the formation of that stem. We perform this search for the n highest correlation values, which gives us n potential stems [2]

Comment 5 — Page 3, can others elaborate on the parameterization of the weights.
Reply — R1 recommended that we elaborate on the choices of the weights, which we answered in page 5 where we argued on our choices in an additional sentence.
Modification — ..., and has the chosen weight; therefore, the set of weights can be seen as a simplified energy function scoring stems. If all the weights are set to 1, $cor(k)$ gives the likelihood of base pairs for a positional lag k. However, the weights can be tuned in order to facilitate the detection of stems containing some types of stems, *i.e.* as G-C interactions are known to be stronger, a larger weight can be used to favor the detection of G-C rich stems.

Comment 6 — Page 3, figure 3 is referred before figure 2. Figures should be numbered in the same order they are cited in the text.
Reply — We renumbered the figures.

Comment 7 — Page 3, Sentence "Linear fold is the fastest..." seems out of context.
Reply — We believe that acknowledging the speed of LinearFold for longer sequences is informative to the reader and fair. Therefore, we slightly change the sentence in order to reflect this.
Modification — The naive algorithm is very fast for sequence up to 10^4; however, Linear-fold is the fastest for the largest sequences.

Comment 8 — R1: Figure 6, What do the notations H,M,B etc denote?
Reply — We enriched Figure 6's caption with the definition of these notations
Modification — The arrows represent the direction to secondary structure types (H=hairpin, I= E=exterior loop, I=interior loop, H=hairpin, B=bulge, S=stacks, M=multi-loop and R=root node).

Reviewer 2

Reviewer 2's Description

Reviewer #2: This manuscript reports a fast method (RAFFT) to generate base-paired stems, ie secondary structure elements, from a nucleotide sequence. This is fine, but isn't the main issue rather the scoring of the stability of the models? A "kinetic Ansatz" is proposed as a means of finding optimal structures. The model used does however not account for possibly significant barriers between substates. The issue is recognized, but not resolved in the manuscript. This is a major simplification which could be expected to have a large influence on the results The basic RAFFT approach performs so-so in terms of accucacy,; when the 50 top-scoring structures are allowed into the game, the chance of finding the "correc" structure increases.
Minor issue: eq 2 is strange. The right hand side is an illegal matrix multiplication.

Comment 1 — eq 2 is strange. The right hand side is an illegal matrix multiplication.
Reply — R2 spotted a missing "=" sign since the equation only shows that X is a 4-rows matrix that is just detailed on the right-hand side of the equation into the $4 \times L$.

Comment 2 — This is fine, but isn't the main issue rather the scoring of the stability of the models? ...The basic RAFFT approach performs so-so in terms of accucacy,; when the 50 top-scoring structures are allowed into the game, the chance of finding the "correc" structure increases.
Reply — In these two comments, we believe that R2 highlighted the fact that our method cannot identify the "correct" answer, i. e. the observed structure. Whether or not the structure observed (or, more precisely, deduced from experimental or evolutionary data) is precisely identified by RAFFT is indeed not possible; however, the goal of our analysis is quite orthogonal to this fact as we demonstrated that the ensemble constructed often contains at least one relevant structure, the "correct" one. This supports the fact that the kinetic partitioning mechanism is relevant. Moreover, the kinetic ansatz is therefore given a relevant set of structures, which support the predicted folding trajectories. As an answer, we highlighted the two points mentioned above in the following sentence.
Modification — The quality of the trajectories predicted by our framework, and therefore, by our kinetic ansatz depends on the quality of the ensemble of structures predicted by the folding component of our method. Consequently, we try here to answer the question:

Comment 3 — The model used does however not account for possibly significant barriers between substates. The issue is recognized, but not resolved in the manuscript. This is a major simplification which could be expected to have a large influence on the results
Reply — As mentioned by R2, we acknowledged the limitation of not accounting for barriers between substates. As an answer to this comment, we added a proposition to account for this limitation in the discussion and conclusion part (page).
Modification — However, further improvements of the algorithm may be investigated:

- the choice of stems is limited to the largest in each positional lag, a greedy choice that may not be optimal. We propose to add stochasticity in the choice of positional lag to keep, such that running multiple times RAFFT, one can overcome some greediness bottlenecks.
- our method constructs parallel pathways leading to a diverse set of accessible structures, but we have not given any thermodynamic-based criterion to identify which are more likely to resemble the native structure. We suggest using an ML-optimized score to investigate the restrained ensemble of structures predicted by `RAFFT`.
- structures connected in the parallel pathways are separated by the formation or unfolding of a single stem. As mentioned above, RAFFT does not account for barriers between structures that the formation of a stem could involve. Therefore, we propose to apply a post-treatment on the folding graph, where the folding path between structures is investigated using the set of valid atomic folding moves (*e.g.* individual base-pair formation).

References

[1] Liphardt, J., Onoa, B., Smith, S. B., Tinoco Jr, I., and Bustamante, C. Reversible unfolding of single rna molecules by mechanical force. *Science 292*, 5517 (2001), 733–737.

[2] Lorenz, R., Bernhart, S. H., zu Siederdissen, C. H., Tafer, H., Flamm, C., Stadler, P. F., and Hofacker, I. L. Viennarna Package 2.0. *Algorithms for Molecular Biology 6*, 1 (2011), 26.
